# NP30 stimulates Th17 differentiation through DC in *Schistosomiasis Japonicum*

**DOI:** 10.1111/pim.12528

**Published:** 2018-04-25

**Authors:** L. Xu, B. Xue, L. Zhou, Z. Qiu, X. Zhang, N. Xu, Q. Tang, J. Zhu, X. Guan, Z. Feng

**Affiliations:** ^1^ Department of Pathology Nanjing Medical University Nanjing China; ^2^ The Key Laboratory of Antibody Technique of Ministry of Health Nanjing Medical University Nanjing China; ^3^ Department of Pathology Northwestern University Evanston IL USA; ^4^ Huadong Medical Institute of Biotechniques Nanjing China

**Keywords:** CD4 T lymphocytes, dendritic cells, schistosomiasis, Th17 cells

## Abstract

The murine monoclonal anti‐idiotypic antibody, NP30, is a potential vaccine candidate against *Schistosoma japonicum*. Previous studies have revealed that NP30 has an immunoregulatory effect, but the underlying mechanism for this effect remains unknown. This study shows that NP30 induces dendritic cell (DC) maturation and increases the production of pro‐inflammatory cytokines. The expression of CD86 and MHC II was upregulated in DCs following stimulation with NP30 in vitro. Moreover, NP30 induced Th17 polarization by increasing the production of IL‐6 and TGF‐β. In vivo, Th17 differentiation was induced by the production of key pro‐inflammatory cytokines, including IL‐6and TGF‐β, from DCs of NP30‐immunized mice. These results indicate that NP30 promotes Th17 polarization through DC activation, preventing serious schistosomiasis.

**Abbreviations**
APC: antigen‐presenting cell
BMDC: bone marrow DC
GAA: gut‐associated antigen
ROR: orphan nuclear receptor
SEA: soluble egg antigen
SSA: soluble schistosomulum antigen
SWA: soluble adult worm antigen

## 1 INTRODUCTION

In China, *Schistosomiasis japonicum* has been one of the most dangerous parasitic diseases for more than 2000 years.^1^ For decades, praziquantel has been the only drug widely used for the clinical treatment and chemotherapy of *Schistosomiasis japonicum* in China. Although the application of praziquantel has greatly reduced the number of infected people, potential resistance against praziquantel limits its use. According to the survey data of 2013, there were still more than 185 000 cases of schistosomiasis and approximately 68 million individuals at risk of infection in China.^2^ Considering the difficulty in blocking the transmission of *S. japonicum* with the sole use of praziquantel, looking for more effective candidates against *S. japonicum* infection is urgent. Recently, many researchers have been interested in the study of the vaccine of schistosomiasis.

Since Niels Kaj Jerne proposed the immune network theory,^3^ it has been widely demonstrated that anti‐idiotypic antibody strategy can be used to mimic cytokines by mimicking its receptor binding epitopes and the kind of anti‐idiotypic antibody the “internal image” of antigen. Taking advantage of their exquisite specificity and high affinity, anti‐idiotypic antibodies have been used as a strong tool in the treatment of infectious diseases and antipathologic vaccine research.^4^ NP30 is the anti‐idiotypic antibody of *Schistosoma japonicum* gut‐associated antigen (GAA), which is a kind of IgM secreted by the hybridoma cells, according to Guan.^5^ Based on the theory of the immune network, NP30 belongs to the family of β‐class anti‐idiotype antibodies, which not only bind to the paratope but also represent a three‐dimensional inversion of the nominal antigen and can therefore be used as surrogate antigens, for example for further immunizations or in ligand‐binding assay applications.^6^In addition to being an “antigen reagent” in the diagnostic assays of *Schistosomiasis japonicum* for years in China, NP30 has also induced a protection rate of 50.46% against the challenge of *Schistosoma japonicum* cercariae.^7^, ^8^ The transfer of NP30 results in smaller granulomas around parasite eggs and lower portal pressure in vivo, which suggested that the anti‐idiotypic antibody had the potential for the treatment of schistosome infection through an immune regulation mechanism. Nevertheless, to date, there are few reports on monoclonal anti‐idiotypic antibodies for the vaccination of schistosomiasis due to the shortage of related research on mechanisms.^5^

Depending on the production of many different associated antigens, *Schistosoma japonicum* stimulates the secretion of some pro‐inflammatory cytokines to induce Th1 and Th2 cells, which play key roles in the infection immune responses.^9^ During the acute stage of *S. japonicum* infection, schistosome antigens induce Th1‐dominant cell‐mediated immune response in the host. During the chronic infection stage, Th1‐type cellular immunity shifts to Th2‐type cellular immunity.^10^ Particularly, some recent studies have revealed that Th17 cells play crucial roles in the pathology in schistosomiasis.^11^ Moreover, in the context of severe egg‐induced immunopathology, this differentiation of Th17 cells stimulates antigen‐presenting cells (APCs) to secret some pro‐inflammatory cytokines.^12^, ^13^ APCs, especially DCs, are useful for studying the mechanisms underlying the immune regulation against schistosomiasis. It has been reported that some anti‐idiotypic antibodies upregulate the coreceptors of DCs and sustain CD4^+^ lymphocyte activation through binding to DCs.^14^ In previous studies, we found that the immunization of NP30 can enhance not only Th2 but also Th1 differentiation, and at the same time, the binding of DC with NP30 was detected. However, the outcome of DC exposure to NP30 and the differentiation of Th17 have not yet been documented. Our hypothesis is that NP30 may stimulate Th17 differentiation through increasing the expression of some particular surface molecules of DCs.

In this study, we detect the expressions of costimulatory molecules on DCs’ cytokine productions and the differentiation of CD4^+^T cell cultured with dendritic cells taken from normal or NP30‐immunized mice. The results indicate the restricted activation state of DCs stimulated with NP30 and production of nonpathogenic Th17.

## 2 MATERIALS AND METHODS

### 2.1 Ethics statement

All experiments were performed in strict accordance with the Regulations for the Administration of Affairs Concerning Experimental Animals, and all efforts were made to minimize animal suffering. All animal procedures were approved by the Institutional Animal Care and Use Committee of Nanjing Medical University for the use of laboratory animals.

### 2.2 Mice, parasites and infection

BALB/c mice, 6‐8 weeks old, were purchased from Comparative Medicine Center of Yangzhou University (Yangzhou, China) and bred in university facilities. All animal experiments were performed in accordance with the Chinese laws for animal protection and in adherence with experimental guidelines and procedures approved by the Institutional Animal Care and Use Committee of Nanjing Medical University for the use of laboratory animals. *Oncomelaniahupensis* harbouring *S. japonicum* cercariae (Chinese mainland strain) were purchased from the Jiangsu Institute of Parasitic Diseases (Wuxi, China).

### 2.3 Preparation of antigens and immunization schedule

A hybridoma cell line secreting an IgM monoclonal anti‐idiotypic antibody‐designated NP30 was obtained from a fusion of SP2/0 and spleen cells of BALB/c mice chronically infected with *Schistosoma japonicum* for one and a half years and identified by screening with immunized rabbit sera against GAA and soluble egg antigen (SEA) of *S. japonicum*. Although GAA is appropriate as a positive control of NP30, it is so difficult to obtain that we did not have enough GAA for all the experiments. Because Sea and GAA have the same antigenicity, NP30 not only reacts with GAA but also cross‐reacts with SEA. Therefore, SEA has been used successfully as a positive control of NP30 for years in our laboratory. NP30 hybridoma cells and irrelevant hybridoma cells used as negative controls were cultured in DMEM supplemented with 10% foetal calf serum and 100 IU/mL of streptomycin and 100 μg/mL of penicillin. Supernatants of both kinds of cells were purified by ammonium sulphate precipitation and HiTrap IgM purification HP (GE Healthcare, Hatfield, United Kingdom). Detoxi‐Gel Endotoxin Removing Gel (Thermo Fisher Scientific Inc., Boston, MA, USA) was used to remove endotoxin. The quality and quantity of purified NP30 were monitored by SDS‐PAGE and A280 absorbance. Antibodies were stored at −70°C until use. The *S. japonicum* soluble egg antigens (SEAs) were provided for use by the Jiangsu Institute of Parasitic Diseases.

BALB/c mice were divided randomly into three groups (two test groups and one uninfected control group) consisting of 24 mice per group. Each mouse was injected intraperitoneally with 100 μL of a solution containing 10 μg of NP30, 100 μL negative control containing 10 μg of irrelevant IgM or 5 μg of SEA. All three groups of mice were immunized three times with a 14‐day interval. Two weeks after the last immunization, each mouse was infected with 12 cercariae of *S. japonicum* through the abdominal skin. Before infection and at 4 or 7 weeks post‐infection, eight mice were randomly chosen from each group and killed for further study. The cells collected from each mouse of one group were pooled together for further experiments.

### 2.4 Cell preparation

#### 2.4.1 Bone marrow‐derived DC (BMDC)

Bone marrow cells were flushed from the femurs and tibias of BALB/c mice, RBC were lysed with Tris‐ammonium chloride buffer, and the remaining cells were cultured at a concentration of 1 × 106 cells/ml in 10 mL of complete‐RPMI 1640 medium containing 10% heat‐inactivated FBS, 100 U/mL penicillin, 100 mg/mL streptomycin with 10 ng/mL GM‐CSF and 5 ng/mL IL‐4 (PeproTech, Rocky Hill, NJ, USA). Two days after seeding, an additional GM‐CSF and IL‐4 were added to cultures. At day 9, nonadherent/semi‐adherent cells were harvested, CD11c^+^ cells were sorted with anti‐CD11c‐Cy5.5 (eBioscience, San Diego, CA, USA) by FACS, and the purity of the sorting was approximately 95%.

#### 2.4.2 DCs from spleen

Spleens taken from NP30‐immunized and uninfected control mice were homogenized with ice‐cold Hanks’ balanced salt solution. After being treated with DNase I (Sigma‐Aldrich, St. Louis, MO, USA) and collagenase type IV (Worthington, Lakewood, NJ, USA), samples were incubated with EDTA for 5 minutes. CD11c^+^ DCs were sorted by FACS.

#### 2.4.3 BMDC‐NP30 cocultures

BMDCs were cultured with negative control (100 μg/mL), NP30 (100 μg/mL), SEA (20 μg/mL) or LPS (1 μg/mL; Sigma‐Aldrich, St. Louis, MO, USA). After 24 hours, the cells and supernatants were collected. IL‐6 and IL‐23 in cell‐free supernatants were measured with a bead‐based cytokine detection assay named FlowCytomix (Bender Medsystems, Vienna, Austria). TGF‐β was detected by a mouse TGF‐beta ELISA Kit (Excell bio, Shanghai, China). The effect of NP30 on DC maturation was determined by measuring expressions of surface molecules by FACS.

#### 2.4.4 DC‐T cell‐NP30 cocultures

Single‐cell suspensions were prepared with the spleens taken from BALB/c mice. RBCs were lysed, and CD4^+^T cells were purified with FITC‐conjugated antibody against CD4 by FACS. Purified CD4^+^ T cells (3 × 10^6^) plus syngeneicDCs (7.5 × 10^5^) that had been treated with NP30, SEA or control for 24 hours were cultured in 6‐well plates in the presence of 1 μg/mL soluble anti‐CD3ε and anti‐CD28 (PeproTech, Rocky Hill, NJ, USA). After 72 hours, supernatants were assayed by Flowcytomix for IL‐4, IL‐5, IL‐6, IL‐23, IL‐17 and IFN‐γ, and TGF‐β was detected by ELISA.

### 2.5 Flow cytometric analysis of NP30 binding to DCs and cell surface molecule expression by DCs

10^6^DCs or CD4^+^T cells were incubated with NP30 for 24 hours. After incubation, cells were reacted with FITC‐conjugated antibodies against IgM (or isotype control antibody) (eBioscience, San Diego, CA, USA) according to the protocol, washed twice and analysed by FACS Calibur flow cytometer. Data were expressed as MFI.

Cells from DC‐NP30 cocultures were stained with anti‐CD11c‐Cy5.5, FITC‐conjugated antibodies against CD80 and MHCII (or isotype IgG control antibody), PE‐conjugated antibodies against CD86 and CD40 (or isotype IgG2aκ control antibody) (eBioscience, San Diego, CA, USA) according to the manufacturer's protocol. After that, the stained cells were gated on the CD11c^+^ population for the analysis of CD40, CD80, CD86 and MHCII with FACS. Data were analysed using FlowJo Flow Cytometry Analysis Software.

### 2.6 Flow cytometric analysis of the proportions of CD4^+^ cells

DCs collected from mice killed at 0 (uninfected control groups were not infected with cercariae), 4 or 7 weeks post‐infection with 12 *S. japonicum* cercariae and vaccination with NP30 or SEA were cultured with CD4^+^T cells for 24 hours. Lymphocytes were stimulated with PMA and ionomycin. Cells were surface stained with anti‐CD3‐APC and anti‐CD4‐FITC and then intracellularly stained with PE‐conjugated antibodies against IL‐17A, IFN‐γ, IL‐4 or isotype IgG2a control antibody for FACS analysis of Th17, Th1 or Th2 cells. Tregs were detected using the Mouse Regulatory T Cell Staining Kit according to the protocols. To identify and evaluate nonpathogenic Th17 cells, the CD4^+^T cells were intracellularly stained with PE‐anti‐IL‐17A Ab and Alexa Fluor 488‐anti‐IL‐10 according to the manufacturer's instructions (eBioscience, San Diego, CA, USA).

### 2.7 Statistical analysis

One‐way ANOVA was used to determine the statistical analysis of the differences between groups. *P* values of <.05 were considered significant and were calculated with GraphPad Prism 5.0.

## 3 RESULTS

### 3.1 NP30 binding to DC in vitro

**Figure 1:**
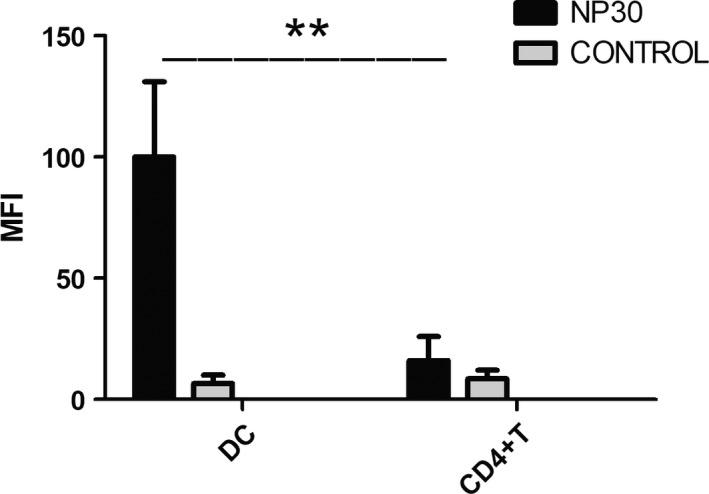
NP30 binding to DCs. DCs and CD4^+^T cells were incubated with NP30 or PBS for 24 h. After incubation, cells were reacted with FITC‐conjugated antibodies against IgM. Cells were analysed by FACS. DCs showed the higher level of NP30 binding and uptake. On the contrary, CD4^+^ T cells did not bind NP30 significantly. The results are shown as the mean of five experiments+standard error of the mean. **P <* *.01 compared to control cells

In a first series of experiments, we evaluated the possible interaction of NP30 with immune cells. To this purpose, DCs and CD4^+^T cells were mixed with NP30 for 24 hours, and after addition of IgM‐FITC, NP30 surface binding and intracellular uptake were determined by cytofluorimetric analysis and expressed as MFI. The results showed that DCs showed the higher level of NP30 binding and uptake. On the contrary, CD4^+^ T cells did not bind NP30 significantly (Figure 1). Binding of NP30 resulted marginal (MFI ranging from 10 to 15) in each cell population evaluated.

### 3.2 CD86 and MHCII expressions on DCs are upregulated following stimulation with NP30 in vitro

**Figure 2:**
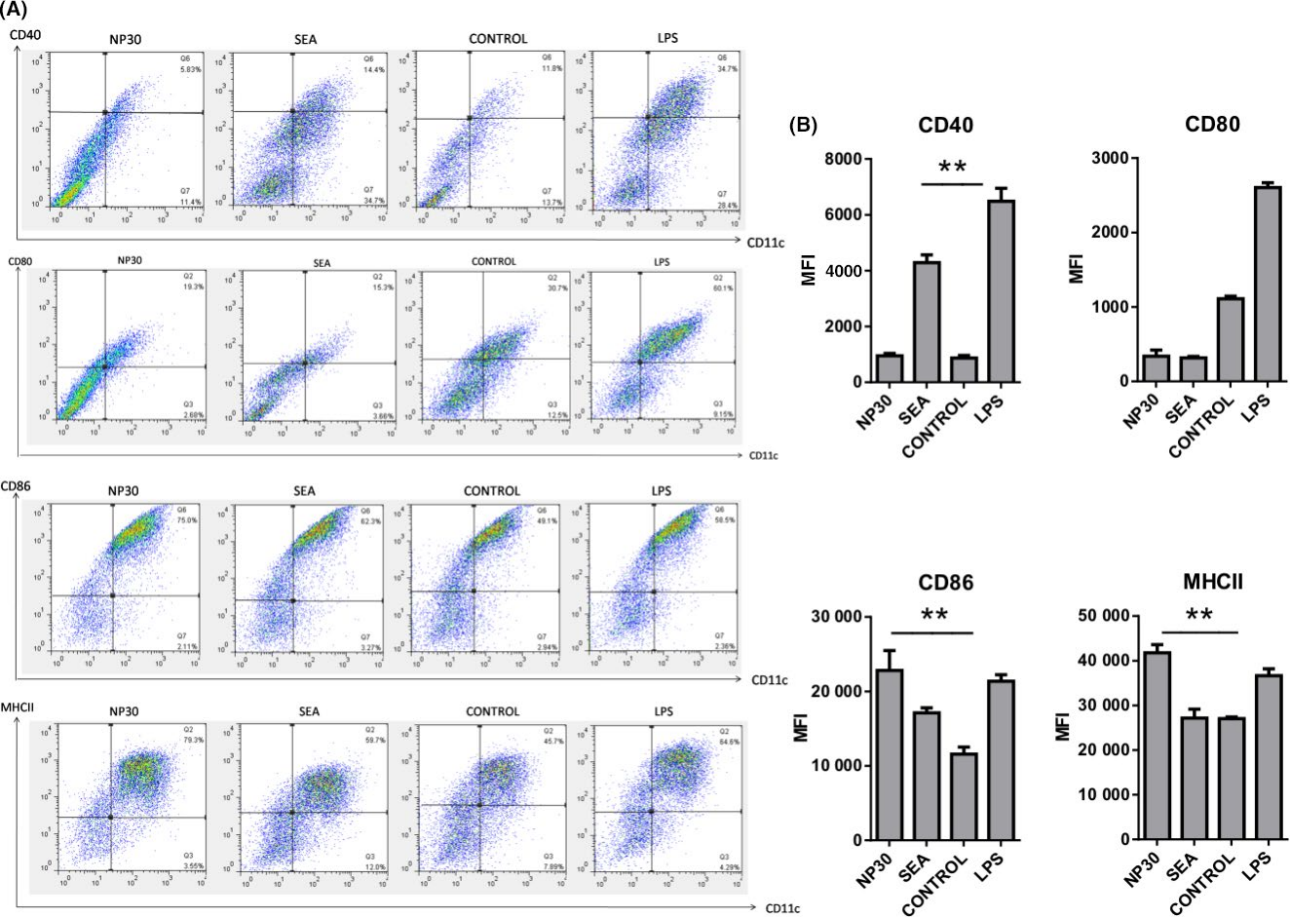
Expression of surface markers on DCs. Bone marrow cells were induced by GM‐CSF and IL‐4 for 9 days, and nonadherent/semi‐adherent cells were harvested as bone marrow DCs. BDMCs were divided into four groups and cultured with NP30, SEA, control and LPS. Twenty‐four hours later, BMDCs were gated with CD11c(A), and the expressions of CD40^+^ CD11c^+^(B), CD80^+^ CD11c^+^(C), CD86^+^ CD11c^+^(D) and MHCII ^+^ CD11c^+^(E) were measured by FACS. BMDCs stimulated with SEA expressed more CD40 than the control group, and NP30 group increased high CD86 and MHCII expressions. The results are shown as the mean of five experiments+standard error of the mean and expressed as the percentage of positive cells. **P <* *.01 compared to control cells

To detect whether NP30 exerts effects on DCs, the expressions of CD40, CD80, CD86 and MHCII on the surface of DCs were examined. DCs’ LPS stimulation was used as a positive control. First, we inspected the morphology of DCs that had been activated in the presence of all four groups. The morphology, density and attachment did not differ in various groups of DCs. The expressions of activation markers (CD40, CD80, CD86 and MHCII) of purified DCs (>95% CD11c^+^) were analysed with FACS. As expected, compared to the control group, DCs’ composure to LPS expressed higher levels of all costimulatory molecules. SEA induced a noticeable increase in CD40 expression, but significant changes in CD80, CD86 or MHC II were not detected. However, DCs stimulated with NP30 have not matured to the same extent as DC^SEA^ and DC^LPS^. DC^NP30^ increased the levels of CD86 and MHC II, while the differences in CD40 or CD80 expressions were not statistically significant (Figure 2). Taken together, with various expressions of DCs’ surface activation markers, the phenotypes of DCs stimulated with SEA and NP30 were consistent with different restricted activations. The differences in the extent of DC activation stimulated by SEA and NP30 may play key roles in the difference in the level of T‐cell proliferation stimulated by DC^SEA^ and DC^NP30^.

### 3.3 Pro‐inflammatory cytokines are produced in response to NP30 in vitro

**Figure 3:**
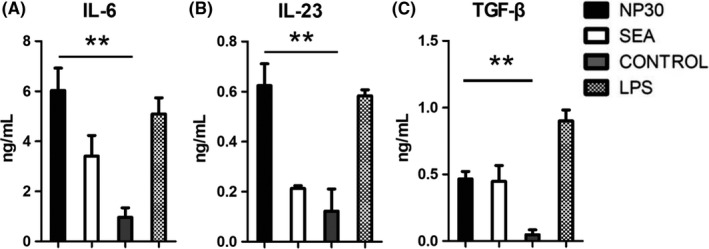
Secretion of Th17 optimal cytokines by DCs. BDMCs were divided into four groups and cultured with NP30, SEA, control and LPS. Twenty‐four hours later, supernatants were collected and secretions of IL‐6(A)and IL‐23(B) were measured with Flowcytomix and TGF‐β(C) was assayed by ELISA. DCs stimulated by NP30 enhance the production of IL‐6, IL‐23 and TGF‐β. The results are shown as the mean of five experiments+standard error of the mean. **P <* *.01 compared to control cells

Only maturation is not sufficient to cause DCs capable of inducing T‐cell differentiation. Another functional property of DCs is the production of inflammatory mediators. Considering the strong relationship of *S. japonicum*‐induced immunopathology with high levels of IL‐17, we detected the optimal Th17 cytokines, such as IL‐6, IL‐23 and TGF‐β, in different groups of DCs. The results showed that DCs in the presence of NP30 or SEA produced much more IL‐6 and TGF‐β than control DCs, and the production of IL‐23 was also higher than control DCs (Figure 3). Because optimal Th17 differentiation and IL‐17 expression were promoted with the combination of IL‐6, TGF‐β and IL‐23, these results suggest that DCs stimulated with NP30 and SEA have the ability to direct DCs towards a Th17 response.

### 3.4 NP30 induces Th17/Th2 cell differentiation in DC‐CD4^+^T cell cocultures

**Figure 4:**
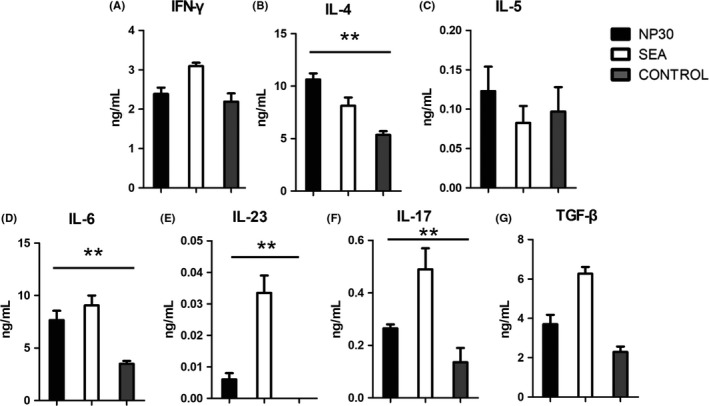
Production of cytokines from coculture of DC‐CD4^+^T cells. CD4^+^T cells were collected from spleens and sorted with FITC‐conjugated antibody against CD4 by FACS. CD11c^+^ BMDCs were collected from the bone marrow of allogeneic mice and sorted with Cy5.5‐conjugated antibody against CD11c by FACS. Purified CD4^+^ T cells (3 × 10^6^) plus allogeneic CD11c^+^ BMDCs (7.5 × 10^5^), which had been treated with NP30, SEA or control, were cultured for 72 h. IFN‐γ(A), IL‐4(B), IL‐5(C), IL‐6(D), IL‐23(E) and IL‐17(F) were assayed by Flowcytomix, and TGF‐β(G) was assayed by ELISA. Compared with the control group, CD4^+^T cells stimulated with DC^NP^ 30 produced much more IL‐4, IL‐6, IL‐23 and IL‐17. The results are shown as the mean of five experiments+standard error of the mean. **P <* *.01 compared to control cells

To examine the effects of NP30 on the ability of DCs to stimulate CD4^+^T cells, BMDCs and syngeneic naïve CD4^+^T cells were incubated together with NP30 or SEA. The cytokine productions of different culture supernatants were analysed after 72 hours. Compared with control DCs, the stimulation of CD4^+^T with DC^NP30^ resulted in more IL‐4, IL‐6 and IL‐17 production, and IL‐23 production was a little high (Figure 4). The observation that DC^NP30^ is a potent stimulator of CD4^+^T cells to produce more IL‐4, IL‐6, IL‐17 and IL‐23 reveals that the differentiation of Th17 and Th2 is induced by DC^NP30^.

### 3.5 NP30 regulates the secretion of pro‐inflammatory cytokines from DCs in vivo

**Figure 5:**
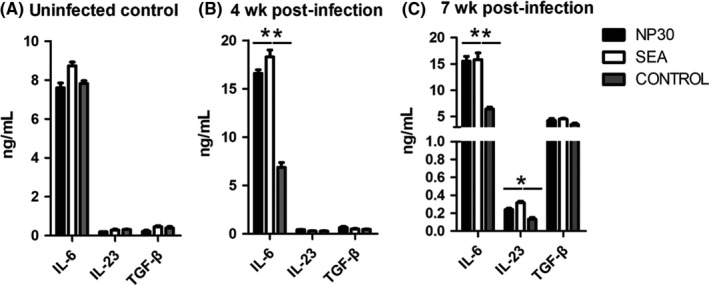
Secretions of optimal Th17 and Th2 cytokines by DCs from mice immunized by NP30 or SEA. Secretions of IL‐4, IL‐6, IL‐23 and TGF‐β by CD11c^+^ DCs isolated from the spleens of mice killed at 0 (uninfected control group) (A), 4(B) or 7(C) weeks post‐infection with 12 cercariae of *S. japonicum* and immunized with NP30 or SEA were assessed. DCs from the 4‐week post‐infection NP30 group produced a high level of IL‐6 and TGF‐β. DCs from 7‐wk post‐infection NP30 group produced more IL‐6 and IL‐23 than the control group. The results are shown as the mean of four experiments+standard error of the mean. *P < .05 and **P <* *.01 compared to control group mice

Thus far, the data reveal that NP30 induces potent Th17 and Th2 responses in vitro, and Th17 and Th2 may be major contributors to the schistosomiasis‐associated pathology of NP30‐immunized mice. To further investigate the functional phenotypes of DCs from infected mice, DCs from mice killed at 0 (before infection), 4 or 7 weeks post‐infection with 12 cercariae of *S. japonicum* after vaccination with NP30, SEA or control were isolated and cultured as described in Materials and Methods. The pro‐inflammatory factors that stimulate Th17 cell activation were detected by Flowcytomix and ELISA. As shown in Figure 5, in supernatants of DCs from mice immunized with NP30 4 and 7 weeks post‐infection, a significantly higher level of IL‐6 was observed compared to the control groups. The production of TGF‐β was also enhanced in the 4‐week post‐infection NP30 group (Figure 5). The increases of IL‐6 and TGF‐β in different periods suggest that repeated vaccination with NP30 preferentially induces DCs to increase Th17 differentiation.

### 3.6 NP30 alters antigen‐specific T‐cell polarization primed by DCs in vivo

**Figure 6:**
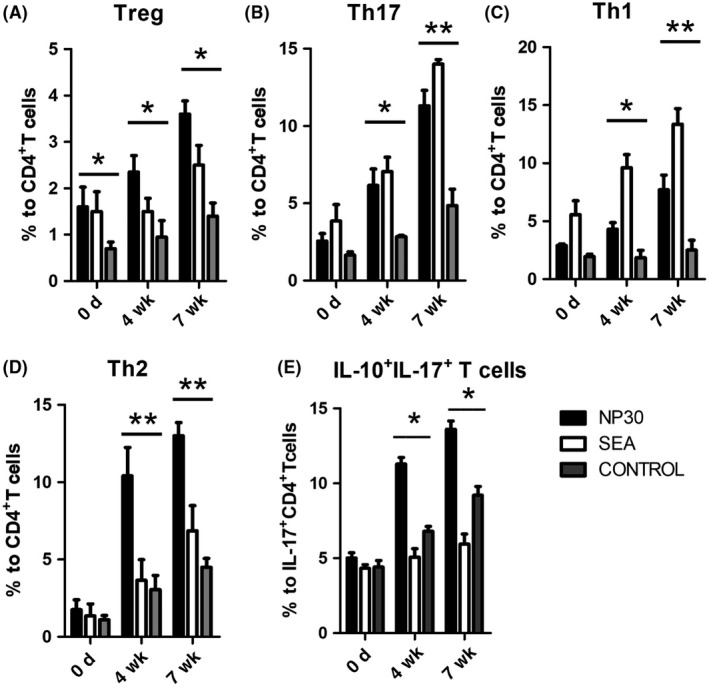
The proportions of Th1, Th2, Th17 and Treg cells from the cocultures of DCs from mice immunized with NP30 or SEA and CD4^+^ T cells. DCs collected from mice killed at 0 (uninfected control group mice were not infected with cercariae), 4 or 7 wk post‐infection with 12 *S. japonicum* cercariae and vaccination with NP30 or SEA were cultured with CD4^+^T cells for 24 h. Lymphocytes were stimulated with PMA and ionomycin. Cells were surface stained with anti‐CD3‐APC and anti‐CD4‐FITC and then intracellularly stained with PE‐conjugated antibodies against IL‐17A, IFN‐c, IL‐4 or isotype IgG2a control antibody for FACS analysis of Th17 (B), Th1(C) or Th2(D) cells. Tregs(A) were detected using the Mouse Regulatory T Cell Staining Kit. The proportion of Th17 cells in splenic CD4^+^T cells increased compared to that before infection (week 0). (E) The percentages of IL‐10^+^ IL‐17^+^ cells to IL‐17^+^ CD4^+^T cells were detected by flow cytometry. The results are presented as the mean of four experiments with the standard error of the mean. *P <* *.05 and **P <* *.01 compared to uninfected control group mice

Naïve CD4^+^ T cells were stimulated with syngeneic DC^NP30^ collected from NP30‐immunized mice sacrificed at 0 (normal group were not infected with cercariae), 4 or 7 weeks after infection. As shown in Figure 6A‐D, the proportion of Th17 cells in splenic CD4^+^T cells increased very slowly during the first four weeks post‐infection compared to that before infection (week 0) and increased rapidly thereafter. Meanwhile, the proportion of Treg cells in the total splenic CD4^+^T cell population showed a continuous increase after infection. Additionally, the proportions of both Th1 and Th2 cells in CD4^+^ T cells also increased (Figure 6A‐D). To determine the role of NP30 in IL‐10 production in nonpathogenic Th17 cells in vivo, we isolated CD4^+^ T cells from mice. As shown in Figure 6E, NP30 immunization induced an increase in IL‐10^+^IL‐17^+^ cells (within total CD4^+^IL‐17^+^ T cells) along with an increase in total IL‐17^+^ cells (Figure 6B and E).

**Figure 7:**
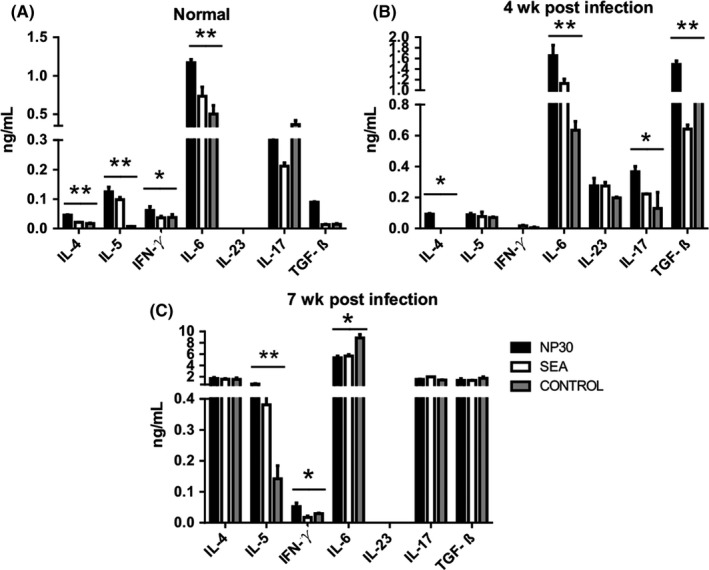
Production of cytokines from coculture of DC‐CD4^+^T cells from mice immunized by NP30 or SEA. Secretions of IL‐4, IL‐5, IFN‐γ, IL‐6, IL‐23, IL‐17 and TGF‐β by DC‐CD4^+^T cell cocultures from mice killed at 0(uninfected control group)(A), 4(B) or 7(C) weeks post‐infection with 12 cercariae of *S. japonicum* and vaccination with NP30 or SEA were assessed. In normal groups, cells from NP30‐immunized mice produced more IL‐4, IL‐5, INF‐γ and IL‐6 than control mice. In the 4‐week post‐infection groups, NP30 group cells secreted high levels of IL‐4, IL‐6, IL‐17 and TGF‐β. In the 7‐week post‐infection groups, cells from NP30‐immunized mice stimulated more IL‐5 and IFN‐γ but less IL‐6 than control mice. The results are shown as the mean of four experiments+standard error of the mean. *P < .05 and **P <* *.01 compared to uninfected control group mice immunized with PBS

The specific cytokines (IFN‐γ, IL‐4, IL‐5, IL‐6, IL‐23, IL‐17A and TGF‐β) released into supernatants were also detected. As shown in Figure 7, the expression levels of IL‐6 and IL‐17 from NP30‐immunized mice were significantly higher than those of the control in the 4‐week post‐infection group. Additionally, IL‐6 was higher in the normal group and lower in the 7‐week post‐infection group than their controls. The IL‐4 and IL‐5 expression levels from the NP30‐immunized mice were higher than those from the control group, and IL‐4 levels were also higher in the 4‐week post‐infection group. Compared with the control group, the expression of IFN‐γ in the NP30‐immunized mice was higher in the normal group and the 7‐week post‐infection group, while the expression of TGF‐β was higher only in the 4‐week post‐infection group. These data suggest that NP30 can regulate the polarization of CD4^+^ T cells through DCs. Although Th17 differentiation was inhibited by the promotion of Th1 and Th2 cells in the normal group, Th17 polarization was primed by DCs in the 4‐week post‐infection group. However, no increase in IL‐23 was detected in any of the groups. These data indicate that NP30 immunization has the ability to induce more IL‐17 secretion induced by DCs at 4 weeks post‐infection (Figure 7).

## 4 DISCUSSION

Currently, the main vaccines of schistosomiasis are nucleic acid vaccines, synthesized peptide vaccine, anti‐id vaccine, recombinant antigen of cDNA and others. Because of the unsatisfactory protection by DNA recombination, the anti‐id method must be used. Previously, we used a one‐step immunization method and hybridoma technique to produce NP30, which was proven as an internal image anti‐idiotypic monoclonal antibody of GAA of *S. japonicum* by ELISA. Our previous studies suggested that NP30 was not only a potential vaccine candidate against *S. japonicum* but also had potential for the treatment of schistosomiasis.^5^ Although we have already noticed that NP30 has the ability to improve Th1 and Th2 differentiation, these results are far from a satisfactory answer to the question of how NP30 produces significant protection to *S. japonicum* **.**

During the past few years, many studies have proven that through the expression of chemokines and the secretion of cytokines, the responses of Th17 cells dominate and play key roles in the host defence in response to *S. japonicum*.^15^, ^16^ Because Th17 has become an important supplement to Th1/Th2 paradigm,^10^ we must ask the question of whether or not NP30 can direct Th17 differentiation.

It is well known that DCs are important in controlling the direction and extent of immune responses through linking innate and acquired immunity. The presentation of Ags by DCs triggers the polarization of naïve T cells (Th0) into different effective T cells. The expression of activation markers on DCs’ surfaces and the cytokines secreted by DCs play key roles in promoting the polarization of Th0 cells into different phenotypes, including Th1, Th2, Th17 or Treg cells.

In the present study, we demonstrated that NP30 binds to DC, possibly produces phenotypic and functional changes on DC, modulating DC capacity to induce an important response of CD4^+^T cells. To further clarify the mechanism that leads to Th17 cell differentiation after exposure to NP30, we used BMDCs, which were cultured with NP30, SEA or LPS in vitro. BMDC exposure to LPS induced an increase in the expressions of costimulatory (CD40, CD80 and CD86) molecules. NP30 enhanced the expressions of CD86 and MHCII even higher than LPS; however, significant differences in CD40 and CD80 expressions between the NP30 treatment group and control group were not observed. Previous articles reported that SEA stimulated a minor increase in MHCII on DC, but not a major enhancement of other molecules of activation, such as CD80, CD86 or CD40.^11^ Furthermore, those antigens come from stages of the *S. mansoni* life circle other than eggs, such as soluble adult worm Ag (SWA) and soluble schistosomulum Ag (SSA), which also fail to mature murine DCs conventionally. Therefore, the results suggest that similar to other antigens of schistosoma, NP30, which is an anti‐id of GAA of *S. japonicum*, induces a likely restricted active DC phenotype with a significantly increased upregulation of the costimulatory markers CD86 and MHC II.

The secretion of cytokines is one of the major attributes of DCs, which plays crucial roles in T‐cell differentiation. Mature DCs produce many different cytokines that can stimulate Th17 differentiation, such as IL‐6, IL‐23 and TGF‐β.^17^, ^18^ In this study, our data suggest that NP30 and SEA‐stimulated DCs secrete much more IL‐6 and TGF‐β than control cells. The activation of Th17 cells has been demonstrated that it is induced from Th0 cells under defined differentiation conditions in numerous in vivo and in vitro studies.^19^ Based on these studies, the polarizing conditions of Th17 cells as follows are widely accepted. In the presence of IL‐6 and TGF‐β, Th17 cells are induced through the expression of the transcription factor RORγt by activating the STAT3 and Smad pathway.^20^, ^21^, ^22^ IL‐23 is important in maintaining the stability of Th17 cells by engaging IL‐23R expressed on activated and memory T cells.^23^, ^24^ In this view, the combination of IL‐6, IL‐23 and TGF‐β promotes the differentiation and expansion of Th17 cells, and our results that DCs exposure to NP30 release more IL‐6, IL‐23 and TGF‐β suggest a strong connection between NP30 and Th17 differentiation mediated by DC. Subsequently, we aimed to clarify the responses of antigen‐specific T cells in the presence of DCs treated with NP30. Our findings showed that naïve CD4^+^T cells were induced to Th17 cells in the interaction with NP30 or SEA‐stimulated DCs, as they produced much more IL‐6 and IL‐17 than control groups.

Some studies have suggested that IL‐4 and IFN‐γ can restrain the differentiation of Th17 cells effectively.^25^, ^26^, ^27^, ^28^ However, our results from animal studies clearly show that along with the polarization of Th17 cells, not only the inducing (IL‐6, TGF‐β) but also the inhibitory (IL‐4) factors of Th17 cell production increased. The findings indicate that antigens (SEA or NP30) from a complicated pathogen, such as *S. japonicum,* introduced complex sets into the host, which could promote a variety of molecules, including not only inhibitory but also inducible factors of Th17 cell differentiation; however, the overall result of the network is an increase in Th17 cell polarization. In other words, the activation of Th17 cells, which has been observed during *S. japonicum* infection, is likely because there are more powerful Th17 inducing factors than the Th17 suppressive factors stimulated by *S. japonicum* antigens. However, the mechanism by which doses of NP30 stimulate Th17 differentiation is still not clear.

As we know, naïve CD4^+^T cells can be activated by some costimulatory signals expressed on the surface of DCs.^29^ Prior studies showed that CD80 and CD86 play key roles in the initiation of immune responses, especially some immune‐mediated diseases. The exposure of costimulatory factors (CD80 or CD86) to TCR (T‐cell receptor) can produce T‐cell differentiation, such as Th1 and Th2. Recently, some studies have revealed that mice deficient in both CD86 and CD80 failed to stimulate the differentiation of Th17, although the mechanisms are still not clear.^30^ However, another report indicated that CD86 but not CD80 enhanced the secretion of IL‐17, and the blockade of CD86 significantly suppressed splenocyte IL‐17 production.^31^ Our data suggest that NP30 stimulates the expression of CD86, but not CD80, in DCs and induces Th17 and Th2 differentiation. Therefore, it is possible that NP30 promotes the differentiation of Th17 by upregulating the expression of CD86.

According to our previous results, NP30 usually induces only mild instead of serious pathological appearances. However, other articles also showed that Th17 cells differentiated by IL‐1‐ and IL‐23‐producing DCs promoted pathological exacerbation and increased the severity of schistosomiasis.^32^ Therefore, we must ask the question of why the Th17 differentiation induced by NP30 does not stimulate serious pathogenicity. Although the differentiation of Th17 cells is responsible for pathological exacerbation in infectious and autoimmune diseases, including schistosomiasis, recent work suggests that only the expression of IL‐17 does not result in pathogenicity automatically. Actually, Th17 cells should be divided into two types based on the circumstances of their induction.^33^ The differentiation of all Th17 cells depends on stimulation with IL‐6 and TGF‐β, but only the presence of IL‐23 induces the pathogenic form, while the absence of IL‐23 gives rise to a nonpathogenic form. Therefore, the expressions of both IL‐17 and IL‐23 in DC‐T cocultures reflect the production of Th17 cells of the pathogenic type and the pathologic effects caused by *S. japonicum*. Following infection with the trematode helminth *Schistosoma mansoni*, severe parasite egg‐induced hepatic granulomatous inflammation as well as pathologic Th17 cell responses driven by dendritic cell (DC) derived more IL‐23. By comparison, mild hepatic immunopathology, egg stimulation of DCs does not result in much IL‐23 production, and pathologic Th17 cells fail to develop.^32^ Our hypothesis is that the differentiation of Th17 induced by NP30 without the expression of IL‐23 produces only mild pathogenic appearances. Therefore, we examined the expression of IL‐23 produced by DCs and CD4^+^T cells from NP30‐immunized mice. In agreement with our hypothesis, our findings indicate that NP30 immunization increased the expression of IL‐17 cells with high increased IL‐23 level. Although the relative contributions of IL‐6, TGF‐βand IL‐23 to Th17 differentiation are confirmed, our data indicate that the Th17 polarization induced by NP30 accompanied only the increased expression of IL‐6 and TGF‐β without much IL‐23. These results confirm that the differentiation of Th17 stimulated by NP30 without the presence of much IL‐23 induces a mild pathogenic form. Additionally, nonpathogenic Th17 cells differentially also express anti‐inflammatory IL‐10.^34^ Although the fraction of IL‐17A‐producing cells within the IL‐10‐producing cell population was relatively small, this fraction represented up to 30% of all IL‐17A producing T cells.^35^ Therefore, we examined the IL‐10‐producing cell population in IL‐17A^+^ CD4^+^ T cells. Consistent with other reports, NP30 immunization induced an increase in IL‐10^+^ Th17 cells along with an increase in total Th17 cells. Taken together, it is possible that the nonpathogenic form of Th17 cell induction enhanced by the immunization of NP30 may not induce but inhibit the severe form of schistosomiasis.

Schistosomiasis is a typical chronic infectious disease. Infection induces the generation of Th1, Th2 and Treg cells, as well as Th17 cells that are involved in the formation of hepatointestinal perioval granulomas. To determine whether NP30 can affect the differentiation of Th17 induced by schistosomiasis, we investigated the differentiation of Th17 cells induced by NP30 from different stages of *S. japonicum* infection in mice. Usually, 4 weeks after infection, as the parasites began to produce eggs, the granulomas continuously develop in the mouse liver. In the present study, with the help of NP30 immunization, the beginning of the deposition of eggs in livers and the formation of granulomas result in the differentiation of Th17 and Th2 that are able to induce IL‐17 and IL‐4 production. Compared with unimmunized control mice, the expression of IL‐4 and IL‐17 primed by DCs from the 4‐week post‐infection group increased when accompanied by NP30 immunization. These results suggest that NP30 is responsible for the increase in the differentiation of Th17 and Th2 promoted by the production of eggs in 4‐week post‐infection mice. However, the mechanism should be clarified in further studies.

In summary, the present study indicates that NP30 stimulates naïve CD4^+^T cell differentiation by modulating DC costimulatory molecule expression and cytokine production, leading to an upregulation of the nonpathologic Th17‐mediated immune response. These findings may increase our understanding of the role of NP30 as a potential therapeutic target.

## CONFLICT OF INTEREST

The authors have declared no conflict of interest.
